# Case Report: Grade 4 pneumonitis occurred after thoracic radiotherapy and dacomitinib in a patient with lung adenocarcinoma

**DOI:** 10.3389/fonc.2025.1436134

**Published:** 2025-02-25

**Authors:** Ailing Liu, Junxu Wen, Kaikai Zhao, Liyang Jiang, Xiangjiao Meng

**Affiliations:** ^1^ School of Clinical Medicine, Shandong Second Medical University, Weifang, China; ^2^ Department of Radiation Oncology, Shandong Cancer Hospital Affiliated to Shandong First Medical University, Jinan, Shandong, China

**Keywords:** non-small cell lung cancer (NSCLC), EGFR mutation, dacomitinib, thoracic radiotherapy, radiation pneumonia

## Abstract

Osimertinib combined with chest radiotherapy has a high incidence of pneumonia, dacomitinib is widely used in clinical practice, but there are no studies reporting the pulmonary safety of dacomitinib in combinating with radiotherapy. Here we report a case of radiation pneumonitis occurring by dacomitinib and thoracic radiotherapy (TRT). The patient was a 55-year-old woman with lung adenocarcinoma. She had received surgery and adjuvant chemotherapy. The patient presented with bilateral intramammary and para-aortic metastatic lymphadenopathy, which was confirmed as metastasis, and subsequently received treatment with dacomitinib. Radiotherapy started after 4 months of dacomitinib. The Clinical Target Volume (CTV) was metastatic lymph nodes area. The prescription dose was 60 Gy/30F. The mean lung dose (MLD), V20, and V5 were 8.16Gy, 16%, and 34.5%. Despite the lung V20 and mean lung dose being exceptionally low, the patient exhibited respiratory symptoms, and a CT chest scan revealed grade 4 radiation pneumonitis two weeks following the conclusion of radiotherapy. The radiotherapy and dacomitinib were discontinued, and immediate initiation of pulmonary anti-inflammatory treatment ensued. The concurrent administration of dacomitinib and RT carries the risk of inducing serious pneumonia. This case highlights the potential risk of severe pneumonia associated with this combination therapy, emphasizing the need for further research to clarify its safety and develop effective management strategies.

## Introduction

Radiation pneumonitis is the most common dose-limiting toxicity for TRT (1). In recent years, lung cancer has ranked first in the global cancer incidence and mortality (2). EGFR activating mutations are present in 15% of NSCLC, and these subgroups are sensitive to EGFR-TKIs (3). Approximately 45% Asian lung adenocarcinoma patients harbor EGFR mutations (4). With the continuous update and development in recent decades, EGFR TKIs are the standard first-line treatment for patients with sensitizing EGFR mutation-positive NSCLC (5). The combination of EGFR-TKI and TRT demonstrates efficacy in treating advanced non-squamous cell lung cancer (6, 7). However, both treatment methods carry potential pulmonary toxicity, and their combination could potentially enhance this toxic effect. For first-generation and third-generation (Osimertinib, aumolertinib) EGFR-TKIs combined with TRT, the incidence of radiation pneumonitis was 33%, 63.6%, and 42.9%, respectively (8–10).

Dacomitinib is a second-generation irreversible EGFR TKI. The phase-3, randomized, open-label, ARCHER 1050 trial demonstrated that first-line dacomitinib significantly improved progress-free survival (PFS) and overall survival (OS) compared with gefitinib in patients with EGFR mutation-positive advanced NSCLC (11).

However, reports of radiation pneumonitis induced by the combination of dacomitinib and radiotherapy are rare. Here, we report a case of grade 4 pneumonitis that occurred following treatment with dacomitinib concurrent with radiotherapy.

## Case presentation

**Figure 1:**
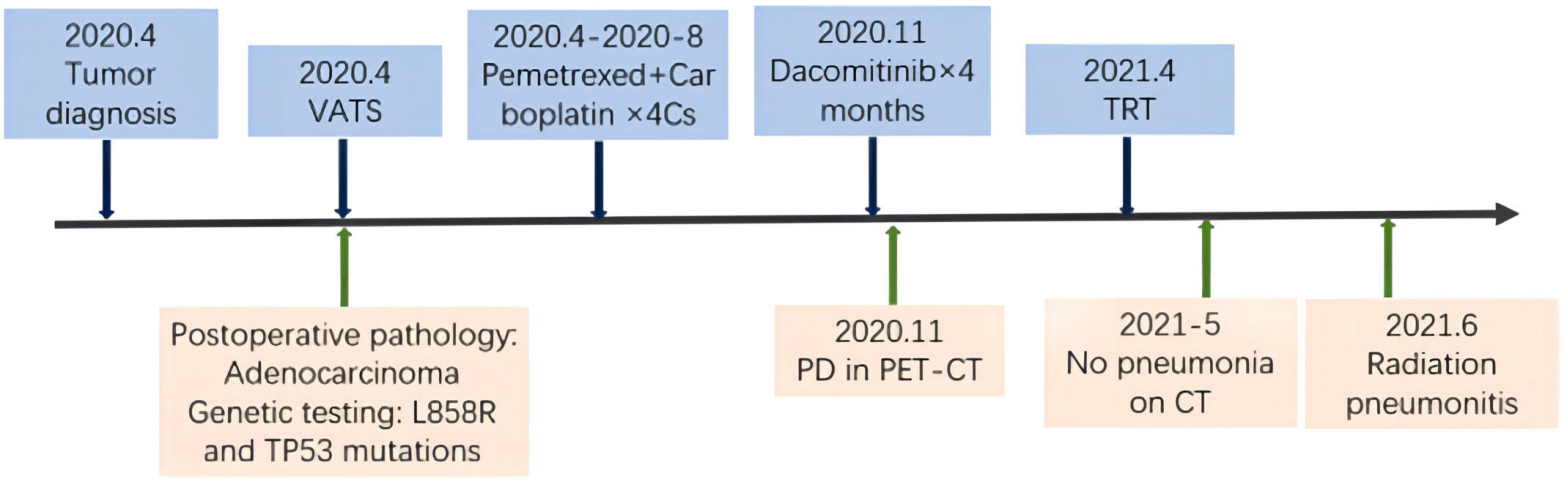
Timeline of the major treatment process and imaging evaluation of the patient since diagnosis. PD means “progressive disease”.

In April 2020, a 55-year-old woman attended to hospital due to routine physical examination; a CT scan showed a space-occupying lesion in the upper left lung lobe’s posterior apical segment with enlargement of the Group 5 and the left hilar lymph nodes (**Figure 1**). A positron emission tomography (PET)/CT scan excluded additional disease localizations. The patient underwent left upper lobectomy with systematic lymphadenectomy by video-assisted thoracic surgery (VATS). Post-surgery pathology showed there to be invasive adenocarcinoma in the upper left lung (1.9*1.6 cm), with Group 5 (3/3) and left hilar (1/5) lymph nodes metastases, and no invasion of the vessels, nerves, pleura or bronchial stump (pT1N2M0 Phase IIIA). Molecular analysis showed EGFR L858R and TP53 gene mutations by next-generation sequencing. The patient received 4 cycles of carboplatin/pemetrexed chemotherapy after surgery, but she refused postoperative radiotherapy and targeted therapy.

**Figure 2:**
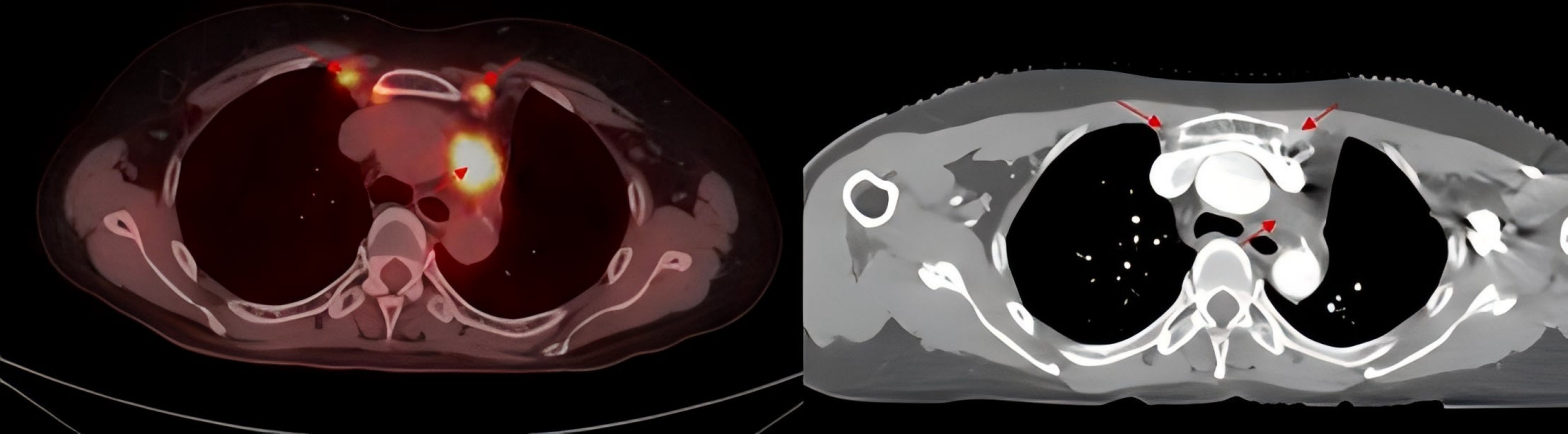
PET/CT imaging shows enlarged bilateral internal mammary and para-aortic lymph nodes and enhanced CT before thoracic radiotherapy.

**Figure 3:**
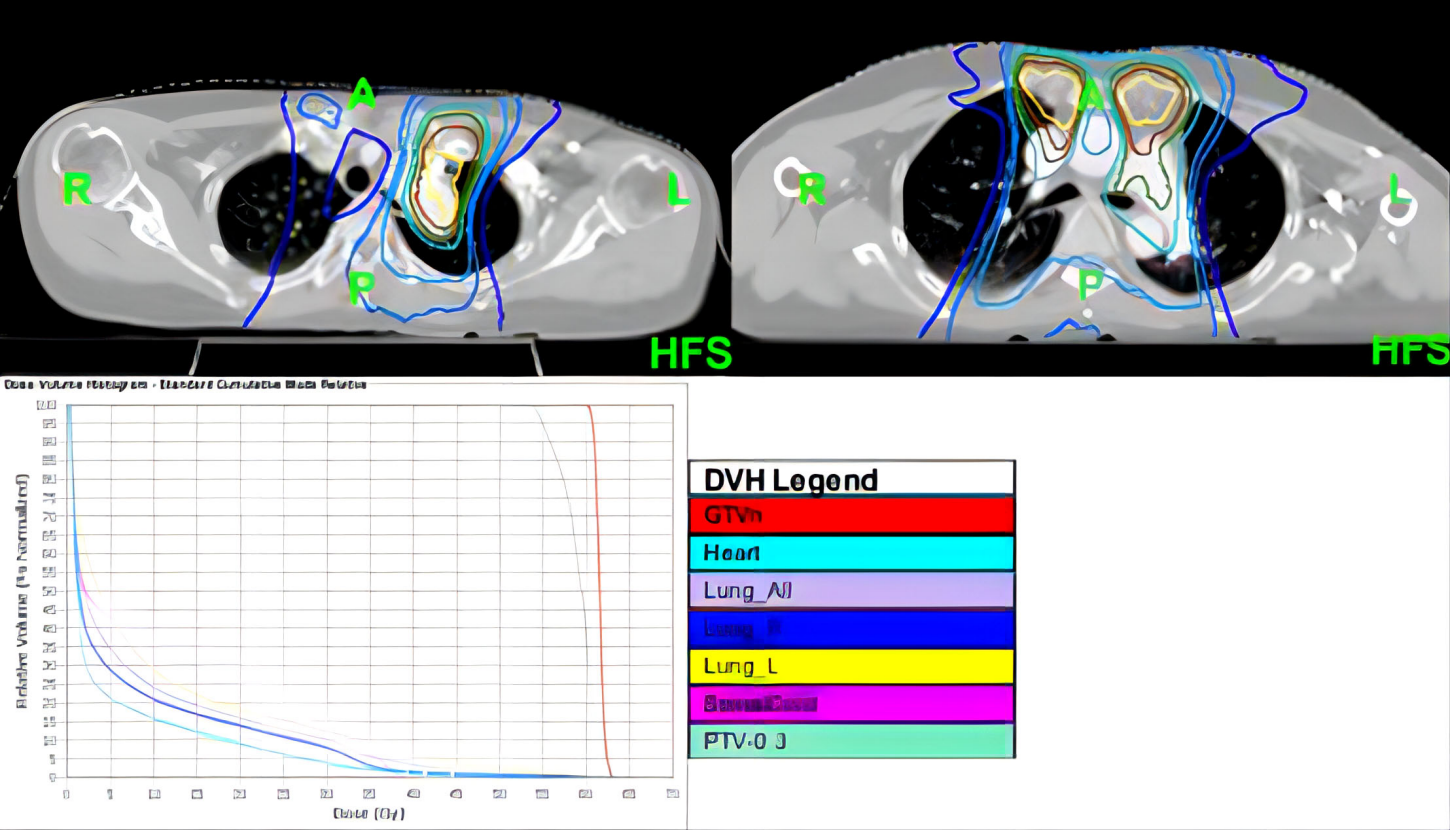
Images showing radiation fields and isodose lines.

**Figure 4:**
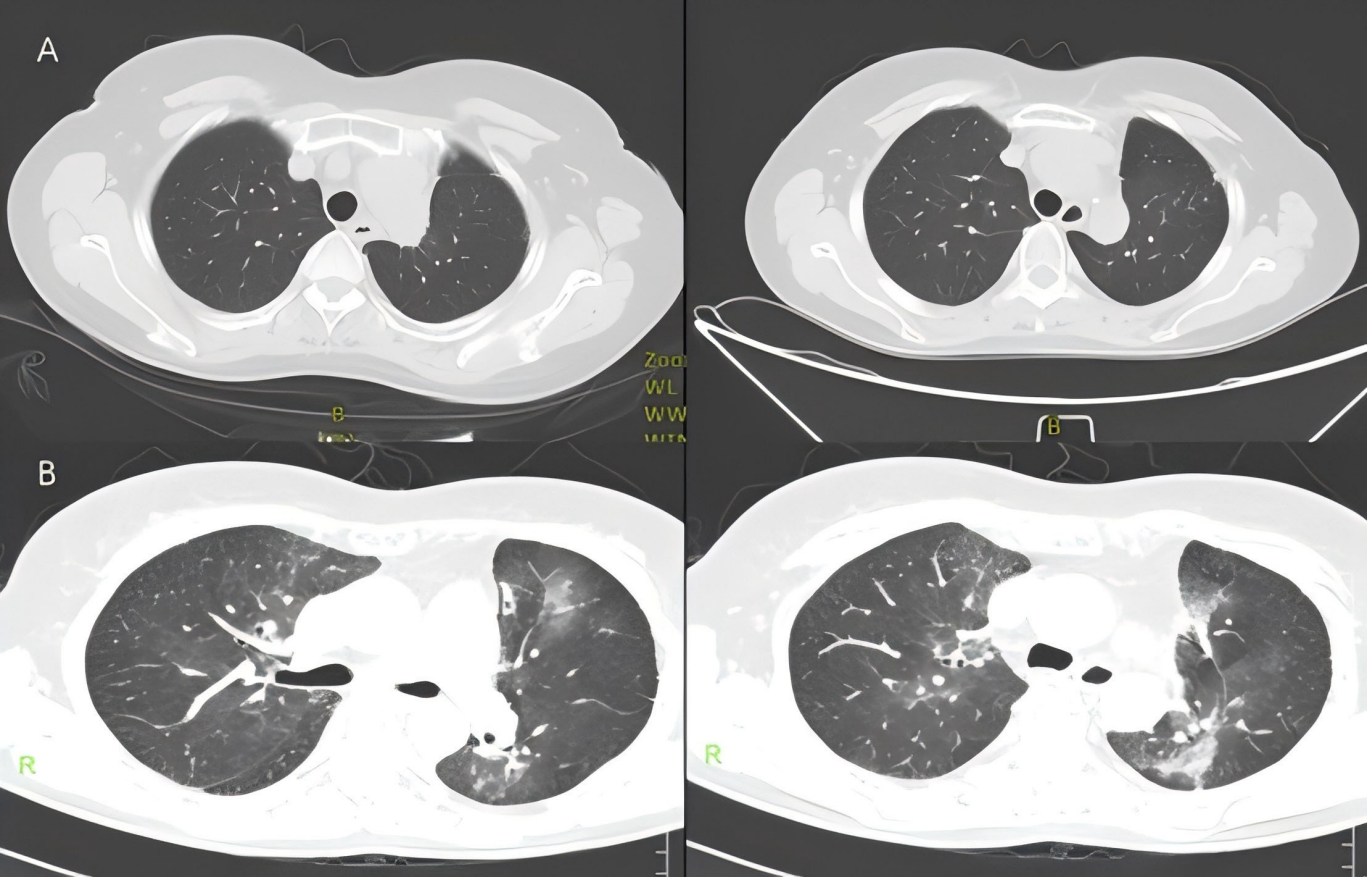
**(A)** Chest CT showed no obvious symptoms of pneumonia on May 8, 2021. **(B)** Chest CT showed radiation pneumonitis on May 29, 2021.

In November 2020, PET/CT scans revealed bilateral internal mammary and para-aortic metastatic lymph node enlargement, indicating tumor recurrence (**Figure 2**). The patient began targeted therapy with dacomitinib. Given the patient belongs to oligometastasis and partial regression were achieved, radiotherapy was performed on the above-mentioned lymph node areas in April 2021. CTV included the enlarged bilateral internal mammary and para-aortic lymph nodes area showed on the PET/CT in November 2020 (**Figure 3**). The prescription dose for the CTV 60 Gy/30F and for the PTV was 54 Gy/30F. The mean lung dose, V5, and V20 were 8.16 Gy, 34.5%, and 16%, respectively. She did not finish radiotherapy in the end because of chest tightness after 27 sessions. The chest CT scan showed no pneumonitis was observed (**Figure 4A**).

Just 2 weeks later, she experienced a slight dry cough and acute shortness of breath. A CT scan showed new patchy ground-glass opacities in the lung bilaterally within the radiation treatment field (**Figure 4B**). Dacomitinib was ceased immediately. However, the patient’s condition continued to deteriorate, resulting in severe respiratory distress, requiring oxygen therapy with a face mask and emergency intervention in the intensive care unit (ICU). To rule out other potential causes of severe respiratory distress, we conducted a comprehensive set of ancillary examinations for the patient. Blood and sputum cultures did not identify any pathogenic microorganisms. Subsequently, the patient underwent bronchoscopy with bronchoalveolar lavage (BAL), and next-generation sequencing (NGS) analysis of the BALF sample did not detect resistant genes, bacteria, fungi, viruses, or other pathogenic microorganisms, suggesting a low likelihood of an infectious etiology. Dacomitinib, as an EGFR-TKI medication, may induce drug-induced pneumonitis; however, the patient’s imaging features (limited to the radiotherapy target area) are more consistent with the characteristics of radiation pneumonitis. The patient had no history of cardiovascular disease, and BNP levels were not significantly elevated, ruling out the possibility of cardiogenic pulmonary edema. This case did not involve the use of immune checkpoint inhibitors (ICIs), nor were there any high-risk factors for immune-related pneumonitis, thus excluding that possibility. Based on the patient’s medical history, the distribution of the radiotherapy target area, and imaging findings, the symptoms and disease course are highly consistent with radiation pneumonitis. According to CTCAE 5.0 criteria, the patient’s CT findings did not meet the typical criteria for grade 4 radiation pneumonitis. However, the patient exhibited severe clinical symptoms, including respiratory distress (hypoxemia), and required ICU treatment, which aligns with the clinical definition of Grade 4 radiation pneumonitis. Given the critical role of clinical symptoms in grading, this case is classified as Grade 4 radiation pneumonitis. The patient was briefly hospitalized and she started treatment with corticosteroids at a dose of 1 mg/kg of IV methylprednisolone (Solumedrol) which was transitioned to oral prednisone. Luckily, the corticosteroid treatment rapidly improved her symptoms. One month later, the patient’s condition improved, breathing difficulties and other symptoms were significantly relieved, and his physical condition gradually recovered. Since then, the patient has continued to receive dacomitinib targeted therapy, and no signs of disease recurrence have been found in the follow-up so far, and the overall prognosis is good.

## Discussion

### Increased risk of radiation pneumonitis

Previous studies have shown that NSCLC patients with EGFR mutations benefit from the combination of RT and targeted treatments, which not only enhances survival and achieves long-term control but also lowers the risk of resistance (12). However, this approach significantly elevates the risk of radiation pneumonitis in some patients. ARCHER 1050 is the first phase-3, randomized, open-label, head-to-head comparison of dacomitinib and gefitinib to show a significant and clinically meaningful improvement in OS (13). Median OS was 34.1 months with dacomitinib versus 27.0 months with gefitinib. In the Asian subgroup, the median OS was even better, reaching 37.7 months. PFS was also superior in dacomitinib arm and the Asian subgroup (11). Dacomitinib improved PFS and OS over gefitinib in Asian patients with EGFR L858R mutation and PFS in Asian patients with EGFR exon 19 deletion mutation. The REFRACT study conducted a retrospective analysis of data from 12 Chinese cancer research institutions, and the results showed that among patients with locally advanced EGFR mutation NSCLC, first-line use of RT+TKI± chemotherapy was associated with better PFS and OS (6). A randomized controlled trial for patients with unresectable stage III NSCLC with EGFR mutations showed that, compared to chemotherapy combined with RT, erlotinib combined with TRT achieved a median PFS of 24.5 months (14). For patients with stage IV EGFR-mutated NSCLC with oligometastatic disease during first-line EGFR-TKI therapy, consolidative local ablative therapy of all residual lesions (including primary tumor, lymph nodes, and metastatic sites as appropriate) is performed. PFS and OS were significantly improved, with median PFS and OS reaching 20.6 months and 40.9 months, respectively (15). Akamatsu H et al. indicated that for patients with oligometastatic locally advanced NSCLC harboring EGFR-sensitive mutations, the median PFS was 18.6 months when treated with gefitinib combined with TRT (16). Compared to first-generation EGFR-TKIs, the use of second-generation (such as dacomitinib) and third-generation (osimertinib) targeted drugs can extend PFS by 5.5 months and 8.7 months, respectively. Patients treated with Osimertinib had their median PFS and OS increased to 18.7 months and 38.6 months, respectively (17). Therefore, for patients with advanced oligometastatic NSCLC with EGFR mutation, the combination of EGFR-TKI and radiotherapy is necessary.

However, the risk of developing radiation pneumonitis also significantly increases. In the RECEL study, the incidence of radiation pneumonitis in the erlotinib combined with radiotherapy (E + RT) group was 16.7%, with mild to moderate (grade 1-2) radiation pneumonitis being the most prevalent, and a few patients developing grade 3-4 pneumonia. In contrast, the incidence and severity of radiation pneumonitis in the etoposide and cisplatin combined with radiotherapy (EP + RT) group were lower. Despite no significant differences in radiation dose or techniques between the two groups, erlotinib may exacerbate radiation-induced damage by inhibiting the repair of EGFR signaling in normal lung tissue (14). The WJOG6911L study reported that the overall incidence of radiation pneumonitis in the gefitinib combined with radiotherapy group was as high as 90%, with 30% of patients discontinuing treatment due to radiation pneumonitis. The median onset of radiation pneumonitis was 92 days, and it correlated with the lung V20 dose distribution. Compared to other EGFR-TKIs (e.g., osimertinib), gefitinib has lower selectivity for normal lung tissue, which may further increase the risk of pneumonia (16). In the study by Zheng et al., the incidence of radiation pneumonitis in patients receiving first-generation EGFR-TKIs combined with radiotherapy was 20%, with most cases occurring around 40 days post-treatment. Although the risk was reduced by limiting the lung V20 dose, radiation pneumonitis remained a significant adverse event during treatment (18). Jia et al. provided the first detailed report of an radiation pneumonitis incidence of 63.6% in patients treated with osimertinib combined with radiotherapy. This phenomenon may be related to osimertinib’s inhibition of alveolar repair and DNA damage repair. Although radiotherapy parameters (e.g., V20) did not significantly increase the risk, osimertinib-induced interstitial lung disease was considered a key factor contributing to the high radiation pneumonitis incidence. Similarly, the study of aumolertinib combined with radiotherapy reported an radiation pneumonitis incidence of 42.9%, with radiation pneumonitis risk closely linked to tumor volume and lung V20 dose. However, the progression-free survival (PFS) was 18.87 months, demonstrating the long-term efficacy of the treatment (9, 10). In previous reports, radiation pneumonitis induced by erlotinib maintenance therapy after stereotactic radiotherapy and concurrent helical CT radiotherapy led to respiratory failure and death in patients (19). In a study by Zhuang et al., 3 out of 24 inoperable stage IIIA-IV NSCLC patients (12.5%) died due to bilateral radiation pneumonitis. Additionally, in a multi-center study, 2 cases (2.9%) of fatal pneumonitis occurred in patients receiving chest radiotherapy and osimertinib (20). It was also noted that among patients with clinically significant pneumonitis, there was an excessively high proportion of central and ultra-central tumors, which is consistent with findings reported by Smith et al., suggesting that central tumors may represent an additional risk factor for the development of pneumonitis in this patient population (21).

These studies indicate that while radiotherapy combined with EGFR-TKI improves survival, it significantly increases the risk of radiation pneumonitis, which remains a major obstacle to this therapeutic approach. Future efforts should focus on optimizing radiotherapy techniques (e.g., proton therapy), selecting less toxic EGFR-TKIs, precise monitoring, early intervention for radiation pneumonitis, and exploring sequential treatment strategies to reduce toxicity and provide safer treatment options for patients.

### Induction of severe radiation pneumonitis

We documented a case of radiation pneumonitis induced by the combination of dacomitinib and radiotherapy, with no detection of other fungal, bacterial, or viral infections throughout the treatment period. Dacomitinib, a second-generation EGFR-TKI, is utilized in the treatment of metastatic NSCLC patients harboring EGFR Del19 or L858R mutations. Functioning as an irreversible inhibitor of the HER family, it blocks effectively signal transduction initiated by these receptors, consequently inhibiting tumor cell growth and survival (22). Common complications associated with dacomitinib use were gastrointestinal and dermatologic complications. These adverse reactions can be overcome through preventive treatment interventions, adjusting medication dosages, and symptomatic management (23). Moreover, a meta-analysis of 16 trials on EGFR TKI treatment in lung cancer patients showed that pneumonia is the most common cause of death related to EGFR TKI toxicity (24). However, EGFR TKIs are generally considered safe, with the risk of severe pneumonia being less than 5%.Suh et al. suggested that the overall incidence of EGFR TKI pneumonitis was 1.12% in patients without prior exposure to EGFR TKI, and 1.13% in retreatment group, but interstitial lung disease or pneumonitis seems to be particularly common in Japanese patients (25). Only 5 patients (0.87%) occurred pneumonia or cellulitis in the Asian subgroup in the ARCHER 1050 trial, while 2 patients (5.0%) experienced interstitial lung disease or pneumonitis in the Japanese subgroup (11, 26). Pulmonary interstitial changes may be the primary cause of radiation pneumonitis after radiotherapy and targeted therapy with the unclear potential internal mechanism (27). As the target of EGFR-TKI, EGFR is primarily expressed in alveolar epithelial cells and is crucial for their proliferation and regeneration (28). EGFR TKIs, by inhibiting EGFR signaling, suppress tumor cell growth and survival. However, during radiotherapy, this suppression may also increase the radiosensitivity of normal lung tissues, resulting in cellular damage. Radiation itself, by causing both direct and indirect DNA damage, intensifies inflammatory responses and promotes cell death (29, 30). Therefore, the combination of EGFR-TKI and TRT may inhibit the self-repair proliferation of alveolar epithelial cells, heightening the risk of lung tissue fibrosis and inflammation. However, the patient in this report experienced Grade 4 radiation pneumonitis after using dacomitinib in combination with RT, and bacterial and fungal infections of the lungs were ruled out. Tsujino et al. showed that V20 greater than 30% is significantly associated with higher incidence of grade 2 or greater radiation pneumonitis in lung cancer patients treated with definitive chemoradiation (31). A risk analysis trial also indicated that V20 > 22% and V30 > 17% are associated with an increased incidence of radiation pneumonitis (32). We estimated that the predictable risk of these patients with high-grade radiation pneumonitis was very low because the V20 was just 16% and the mean lung dose was 8.16 Gy with a small lung volume. We therefore hypothesize that radiation and dacomitinib may have had a synergistic effect in causing severe lung disease in our patient.

### Management of radiation pneumonitis

Radiation pneumonitis is a common side effect of thoracic radiotherapy. Close monitoring of patients’ symptoms, signs and laboratory tests is an important management tool for early detection and prevention of radiation pneumonitis. The primary clinical manifestations of radiation pneumonitis are dyspnea, dry cough, hypoxemia, and mild fever; in severe cases, radiation pneumonitis may result in pulmonary dysfunction and potentially fatal outcomes. When the corresponding symptoms of pneumonia appear, it is necessary to be vigilant, and early intervention should be necessary to avoid further morbidity and even death in these patients. The typical radiologic changes of radiation pneumonitis include ground-glass opacity, diffuse haziness, infiltrates or consoli3dation in the irradiated lung that conform to the shape and size of the radiation fields (33). The bronchoscopy is also very important as it helps obtain direct information about pulmonary parenchymal abnormalities and excludes infectious pneumonia and disease progression.

Despite the high prevalence and incidence of radiation pneumonitis, there are currently no consensus guidelines for its clinical diagnosis, management, and follow-up. Recently, an international Delphi consensus study on the optimal treatment of radiation pneumonitis, conducted by an expert panel comprising oncologists and pulmonologists with expertise in thoracic oncology, reached some preliminary agreements regarding the clinical diagnosis, management, and follow-up of radiation pneumonitis (34). The primary goal of radiation pneumonitis treatment is to alleviate inflammation, and interventions are typically reserved for symptomatic patients. Treatment options include nonsteroidal anti-inflammatory drugs (NSAIDs) or inhaled corticosteroids. Corticosteroid therapy should be tailored based on the severity of the patient’s condition. The usual initial dose is 60 mg of prednisone or 0.5–1 mg/kg, while severe cases may require intravenous administration of 1 mg/kg of methylprednisolone (35). Treatment decisions should account for symptom severity, radiological findings, and concurrent therapies. For oral corticosteroids, it is recommended to maintain the initial dose for at least 2 weeks, followed by a taper of 10 mg per week while monitoring for symptom recurrence. One study indicated that the tapering process generally spans 4–8 weeks. In cases of symptoms worsening, a slower tapering strategy may be employed. Prednisone is frequently used in clinical practice due to its suitability for gradual tapering; compared to dexamethasone, prednisone is more manageable for achieving this process (36). For patients intolerant to corticosteroids, azathioprine and cyclosporine can be used (37). During this period, patient symptoms should be closely monitored, with adjustments to or pauses in the administration of pertinent targeted therapies as needed. Patients with symptomatic Grade 2 and Grades 3-4 radiation pneumonitis are prone to pulmonary infections, and it is advised to start empirical anti-infection treatment early, especially to prevent pneumocystis pneumonia (38). To assess a patient’s recovery from radiation pneumonia, it is recommended to monitor imaging changes through regular high-resolution CT (HRCT) follow-up. The first imaging review should be conducted within 1 month after the end of radiotherapy, and follow-up should be conducted every 3 months thereafter. For patients with extensive lesions or target areas near the center of the lung, the follow-up interval can be shortened, and the follow-up interval can be gradually extended after the symptoms improve significantly. During imaging follow-up, attention should be paid to the absorption of ground glass shadow and solid shadow and whether irreversible fibrosis lesions occur. Gradually reduce glucocorticoids when imaging is stable or improved; Secondary infection or drug toxicity should be vigilant if the lesion is found to be worsening or new, and if conditions permit, the activity and extent of radiation pneumonia can be further assessed by functional imaging techniques such as PET-CT, especially if tumor progression needs to be ruled out. In addition, long-term sequelae need to be paid attention to, radiation pneumonia can progress into irreversible pulmonary fibrosis. In a randomized, Phase II, placebo-controlled trial, it was demonstrated that adding nintedanib to a progressively tapering dose of prednisone can mitigate pulmonary deterioration (39).The prognosis for radiation pneumonia is very good if it is diagnosed and treated accurately and timely before progressing to fibrosis. Although recovery is relatively slow, clinical symptoms can be completely alleviated. Targeted therapy combined with TRT has been proven to provide survival benefits, but it also increases the risk of radiation pneumonitis. Recent studies have shown that for patients receiving both EGFR-TKI and radiotherapy, overlapping treatment duration is an independent risk factor for radiation pneumonitis, suggesting that reducing overlap time can decrease the incidence of radiation pneumonitis (40). Peng et al. pointed out that in patients with stage IV NSCLC undergoing EGFR-TKI and stereotactic radiotherapy, only 6.67% developed grade 2 pneumonia, with no observations of grade 3 or higher levels of pulmonary toxicity (41). Therefore, future research should focus on optimizing the timing and method of combined treatments, investigating the choice of radiotherapy methods, the etiology of radiation pneumonitis, and its targeted treatment strategies. Simultaneously, strengthening preventive measures in clinical practice is necessary to reduce the incidence and severity of radiation pneumonitis.

## Conclusion

To our best knowledge, this is the first case reported of radiation pneumonitis caused by dacomitinib and concurrent radiation. Although the relationship between radiation pneumonitis and dacomitinib remains unclear, our report indicate that oncologists should consider the risk of serious radiation pneumonitis during treatment with dacomitinib in patients who receive thoracic radiotherapy.

## Data Availability

The original contributions presented in the study are included in the article/supplementary material. Further inquiries can be directed to the corresponding authors.
